# The Clinical Usefulness of Taiwan Bivalent Freeze-Dried Hemorrhagic Antivenom in *Protobothrops mucrosquamatus*- and *Viridovipera stejnegeri*-Envenomed Patients

**DOI:** 10.3390/toxins14110794

**Published:** 2022-11-15

**Authors:** Chih-Chuan Lin, Chia-Pang Shih, Chia-Cheng Wang, Chun-Hsiang Ouyang, Chien-Chun Liu, Jau-Song Yu, Chih-Hong Lo

**Affiliations:** 1 Department of Emergency Medicine, Linkou Chang Gung Memorial Hospital, Taoyuan 333, Taiwan; 2 College of Medicine, Chang Gung University, Taoyuan 333, Taiwan; 3 Department of Nursing, Yuanpei University of Medical Technology, Hsinchu 300, Taiwan; 4 Department of Traumatology and Emergency Surgery, Chang Gung Memorial Hospital, Taoyuan 333, Taiwan; 5 Molecular Medicine Research Center, Chang Gung University, Taoyuan 33302, Taiwan; 6 Department of Cell and Molecular Biology, College of Medicine, Chang Gung University, Taoyuan 33302, Taiwan; 7 Liver Research Center, Chang Gung Memorial Hospital, Linkou, Taoyuan 33305, Taiwan; 8 Department of General Surgery, Chang Gung Memorial Hospital, Taoyuan 333, Taiwan

**Keywords:** snake antivenom, snake venom, *Protobothrops mucrosquamatus*, *Viridovipera stejnegeri*

## Abstract

Snakebites from *Protobothrops mucrosquamatus* (Taiwan habus) and *Viridovipera stejnegeri* (green bamboo vipers) account for the most venomous snakebites in Taiwan. The bivalent freeze-dried hemorrhagic (FH) antivenom is employed to treat these two snakebite patients without a strict clinical trial. We evaluated the clinical usefulness of Taiwan bivalent freeze-dried hemorrhagic (FH) antivenom in Taiwan habu- and green bamboo viper-envenomed patients. We checked ELISA- based serum venom antigen levels before and after FH antivenom to evaluate FH’s ability to neutralize patients’ serum snake venom and its usefulness in reducing limb swelling after snakebites. Patients who had higher serum venom antigen levels had more severe limb swelling. Of the 33 enrolled patients, most of their snake venom antigen levels were undetected after the appliance of antivenom. Most enrolled patients (25/33) had their limb swelling subside within 12 h after antivenom treatment. The failure to reduce limb swelling was probably due to an inadequate antivenom dose applied in more severely envenomated patients. Our data indicate the feasibility of the FH antivenom in effectively eliminating venom and resolving the affected limb swelling caused by Taiwan habu and green bamboo viper bites.

## 1. Introduction

Taiwan, located in the subtropical region, is a suitable environment for many snake species. Among them, six venomous snake species are recognized as medically relevant: Taiwan habu (*Protobothrops mucrosquamatus)*, green bamboo viper (*Viridovipera stejnegeri*), hundred-pace snake (*Deinagkistrodon acutus*), Russell’s viper (*Daboia russelii*), Taiwan cobra (*Naja atra*), and Taiwan banded krait (*Bungarus multicinctus*). With a nationwide annual incidence of snakebites of 40.49 per million persons, Taiwan habu and green bamboo viper snakebites account for two-thirds of the envenomation in Taiwan [1]. The major venom components found in Taiwan habu and green bamboo viper were C-type lectin, snake venom serine proteinase, venom metalloproteinase, and phospholipase A_2_, commonly identified in Viperidae snake venoms [2]. Venom metalloproteinase plays a leading role in the prominent local inflammatory response of snakebite envenomation with edema and local or systemic hemorrhage [3]. Therefore, these two kinds of snakebites are often classified as the hemotoxic type of toxicity; however, unlike other Viperidae snakebites, the Taiwan habu- and green bamboo viper-envenomated patients had their symptoms and signs typically characterized by limb swelling, but systemic bleeding or coagulopathy are very rare [4]. On the contrary, in patients with severe limb swelling, surgical intervention such as debridement, fasciotomy, or graft may be needed [5]. Therefore, reducing limb swelling after Taiwan habu or green bamboo viper bites is crucial in treating envenomated patients.

Even without approval in the strict process of clinical trials, antivenom remains the mainstay of treatment for snakebite envenomation [6,7]. The Vaccine Center of the Center for Disease Control of Taiwan developed the F(ab′)_2_ horse immunoglobulin G antivenoms in the 1980s [8,9,10]. The bivalent freeze-dried hemorrhagic (FH) antivenom is used to treat Taiwan habu and green bamboo viper bite patients. Not surprisingly, clinical trials of the effectiveness of the FH antivenom were omitted from the development pathway. In the medical literature, there are only observational or retrospective studies regarding the usefulness of the FH antivenom in treating the Taiwan habu and green bamboo viper snakebites patients so far [4,5,11,12]. The current treatment guideline (provided by the Taiwan poison control center) for FH antivenom is based mainly on expert opinion and the results of in vitro animal studies only [13]. Thus, there is a need to assess the clinical usefulness of FH antivenom. However, it seems impractical to perform the standard clinical trial of the clinical usefulness of FH antivenom at present. We had two aims in this study. The first aim was to explore the association between serum venom antigen levels and clinical severity. The second aim was to investigate the clinical usefulness of Taiwan bivalent-freeze-dried hemorrhagic antivenom in Taiwan habu and green bamboo viper envenomed patients. Previously, we had already developed an ELISA-based venom detection method [14]. By the measurement of serum venom antigen levels, we can achieve the above two study aims.

## 2. Results

### 2.1. Patients’ Characteristics

Of the 33 enrolled snakebite-envenomed patients, males accounted for most of this study population (n = 17, 52%), with a mean age of 61.4 ± 15.8 years old. There were 14 and 19 patients envenomed by Taiwan habu and green bamboo viper, respectively (Table 1). Most patients were admitted to our ED within 2 h (range: 0–16 h) after the bites. The degrees of the clinical severity of the maximum limb swelling ranged from mild to severe in both snakebite patients. There was no statistical difference between Taiwan habu- and green bamboo viper-envenomated patients (*p* = 0.31), except that there was only one patient with a moderate degree of severity in the Taiwan habu snakebites patients.

All patients received antivenom treatment. Most of the patients received only 1–2 vials of antivenom. There were 9 (64.3%) and 16 (84.2%) Taiwan habu and green bamboo viper snakebites patients whose limbs swelling subsided in 12 h, respectively. There were no statistical differences in age, gender, ED admission/referral, patients’ presentation times, laboratory variables such as white cell count, segment, platelet, prothrombin time, activated partial thromboplastin time, the degree of initial and maximum limb swelling, the time interval of progression, and antivenom dosage between Taiwan habu and green bamboo viper snakebites patients (Table 1).

### 2.2. Serum Venom Antigen Levels and Clinical Severity of the Envenomated Patients

The blood samples of pre-antivenom serum venom antigen levels were collected immediately after ED admission. Because referral patients already received antivenom in the referral hospital, blood samples of post-antivenom were collected in 7 Taiwan habu and 15 green bamboo viper referral snakebite patients. Pre-antivenom serum venom antigen levels of Taiwan habu and green bamboo ranged from 0.39 to 25.1 ng/mL, and 0 to 891.1 ng/mL, respectively. The pre-antivenom serum venom antigen levels (median, IQR) were 12.1 (17.9) and 28.2 (130.2) ng/mL for Taiwan habu and green bamboo viper (Table 1).

Table 2 reports the clinical severity and its relationship with serum venom antigen levels by Spearman correlation. In Taiwan habu and green bamboo viper snakebite patients, the clinical severity of maximum limb swelling was positively related to the pre-antivenom serum venom antigen levels (Spearman correlation coefficient (r_s_) = 0.93, *p* = 0.0007; r_s_ = 0.71, *p* = 0.003, in Taiwan habu and green bamboo viper, respectively). There was also a correlation between the maximum limb swelling (the most extended length of the envenomated limbs extended observed in the event) and the use of the total antivenom doses both in Taiwan habu (r_s_ = 0.70, *p* = 0.006) and green bamboo viper (r_s_ = 0.70, *p* = 0.0009) snakebite patients. This was reasonable since more severe patients need more antivenom to treat them. However, there was no statistical association between the degree of initial limb swelling and pre-antivenom serum venom antigen levels.

### 2.3. The Ability of Antivenom to Eliminate Snake Venom in Snakebite Patients

**Figure 1:**
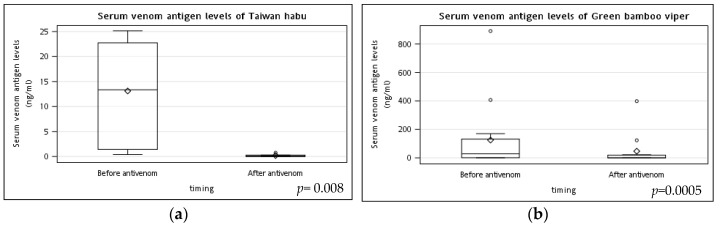
The changes in serum venom antigen levels before and after the antivenom treatment of Taiwan habu and green bamboo viper snakebite patients. Blood samples for pre-antivenom serum venom antigen levels of (**a**) Taiwan habu and (**b**) green bamboo viper bite patients were obtained just before antivenom administration (about 2 h after snakebites). Other blood samples were collected six hours after administering antivenom to detect the post-antivenom serum venom antigen levels. Most patients had their serum venom antigen levels almost undetected after administering antivenom. *p* values were performed by the Wilcoxon signed-rank test. (◊ mean; ○ outlier).

Figure 1 demonstrated the changes in serum venom antigen levels before and after the antivenom treatment of Taiwan habu and green bamboo viper snakebites patients. At 6 h after antivenom therapy, venom antigen levels (median (IQR)) were significantly decreased after antivenom in both Taiwan habu (12.1 (17.9) vs. 0.0 (0.2), *p* = 0.008) and green bamboo viper (28.2 (130.2) vs. 0.0 (15.4), *p* = 0.0005) bite patients. Furthermore, most of the venom levels of Taiwan habu and green bamboo viper snakebites patients (4/7, 57.1%; 7/12, 58.3%, respectively) had decreased to undetectable levels.

### 2.4. The Clinical Usefulness of Antivenom in Treating Snakebites Patients

Most enrolled patients (25/33, 76%) had their limb swelling subside within 12 h after antivenom treatment (Table 1). Patients whose limb swelling subsided within 12 h did not have rebound limb swelling. To further explore why some of the patient’s failure in limb swelling subsided within 12 h after antivenom treatment, we divided the patients into two groups. In Group A (n = 25), the patients had their swelling subside within 12 h after receiving antivenom, and in group B (n = 8), the patients’ swelling did not subside within 12 h after receiving antivenom (Table 3). There were no statistical differences found in the age, gender, patients’ presentation time, laboratory variables such as white cell count, segment, platelet, prothrombin time, activated partial thromboplastin time, the pre-antivenom antigen levels, the time interval of progression, time interval of progression, and antivenom dose between group A and group B. The only difference between these two groups was the degree of maximum limb swelling. Half of the group A patients had their maximum swelling reach a mild degree, while there was no mild degree of swelling in the patients but a moderate to severe degree of limb swelling in the group B patients. The group B patients had their maximum swelling up to the elbow or near the knee joint with the severity of moderate to severe. They received only 1–2 vials of antivenom, and their swelling did subside within 12 h.

**Figure 2:**
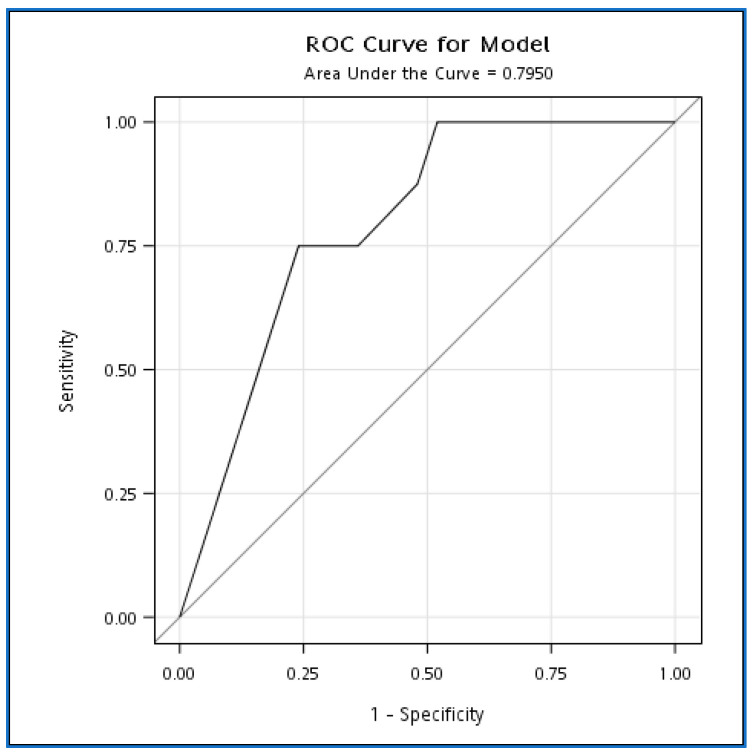
ROC curve for the effect of maximum swelling on swelling subsiding within 12 h.

On the contrary, group A patients had less clinically severe severity compared to group B but with a similar antivenom dose. Their limb swelling subsided within 12 h. Thus, we might conclude that an inadequate antivenom dosage was why group B had their limb swelling not subside within 12 h after antivenom. We perform a logistic model to explore the impact of maximum swelling on subsiding in 12 h. The logistics model showed an increase of 1 degree in the maximum swelling multiplied by the odds of swelling not subsiding in 12 h by an odds ratio of 1.5 (95% CI 1.0–2.0, *p* =0.03), and the area under the curve was 0.795 (95%CI: 0.635~0.955) (Figure 2).

### 2.5. Patients’ Outcome

There was no mortality and no surgical intervention such as debridement, fasciotomy, or graft needed in all patients. All patients receiving antivenom were discharged in good condition after a median of 3 (2–6) days of hospital stay. There was not any allergic effect noticed during antivenom therapy. All patients were followed up at least once in the outpatient clinic one week after ED or ward discharge. More instances of outpatient clinic follow-up were determined depending on the patients’ clinical conditions. All patients were in good condition and no serum sickness symptoms were observed at the follow-up visit one week after discharge. All patients had no sequelae or complications remaining.

## 3. Discussion

Despite the well-accepted use of antivenoms to treat venomous snakebite patients, the routine use of antivenoms has never been formally evaluated in clinical studies [6,15]. Although good immunogenicity was demonstrated by the preclinical assessment of the FH antivenom [10], there were only observational or retrospective studies regarding using the FH antivenom in the treatment of Taiwan habu and green bamboo viper snakebites. To the best of our knowledge, this is the first prospective, observational cohort study to address the clinical usefulness of FH antivenom. In this study, we demonstrated that patients’ serum venom antigen levels were correlated to their clinical severity of envenomation. The FH antivenom had a good ability to bind and eliminate serum venoms in Taiwan habu and green bamboo viper snakebite patients. Under sufficient antivenom dose, limb swelling can be reduced within 12 h. Thus, the clinical usefulness of the FH antivenom in treating local envenomation in snakebite patients might be determined.

### 3.1. Serum Venom Antigen Levels, Clinical Severity, and Antivenom Dose Suggestion

Theakston et al. [16] proposed using an ELISA to detect venom antigens in biological fluids in epidemiological studies, diagnosis envenomation, and pharmacological assessment of antivenoms. Since then, venom antigen levels determined by sandwich ELISA tests have been applied to assess clinical envenomation severity grading. Serum venom antigen levels following bites correlated with the severity of the envenomation in French viper bites (*Vipera aspis*, *Vipera berus,* and *Vipera ammodytes*) and *Bothrops lanceolatus* bites [17,18,19]. In this study, we confirmed that the higher serum venom antigen levels were associated with higher clinical severity in Taiwan habu and green bamboo viper snakebite patients. As a previous report mentioned, the serum venom antigens levels determined during the first four hours following the bite correlated with the severity of the envenomation when the symptoms were determined at their worst [17]. In our study, as in the case of *Bothrops lanceolatus* bites, serum venom antigen levels increased with the grade of severity based on the presence of local signs [19]. Based on the above observation and study findings, we may imply that the ELISA test is a valuable and predictive tool for clinically grading viper envenoming based on predominate local signs.

Besides, the patient’s maximum limb swelling was also correlated to the use of the total antivenom doses both in Taiwan habu and green bamboo viper snakebites patients. This was reasonable since more severe patients need more antivenom to treat them. Therefore, based on the above observation, we could determine the antivenom dose according to serum concentrations. However, there was a small patient number in this study, so it is not suitable to develop such suggestions just based on serum venom antigen levels by using a regression equation to analyze the suggested antivenom doses. Nevertheless, we thought it was reasonable since serum venom antigen levels following the bite correlated with the maximum severity of the envenomation; we thought we could explore the trends in our study and make some suggestions based on our observations.

In Table 4, we described the detail of the antivenom dose, the clinical severity of maximum swelling, and whether swelling subsided within 12 h after antivenom application. Then, we deduced the appropriate dose suggestion for giving antivenom to Taiwan habu/green bamboo viper snakebite patients.

For example, in Taiwan habu-severely envenomated patients, there were eight patients treated with different vials of antivenom. Among them, although four patients received 2–3 vials of antivenom, their limb swelling did not subside within 12 h; however, there were also four patients with the same severity of envenomation who received 1–3 vials of antivenom whose limb swelling subsided within 12 h. Therefore, we suggested that at least 2–3 vials of antivenom should be used to relieve limb swelling in severely envenomated patients.

It is almost difficult to determine the maximal swelling at the moment that patients come to the ED for help after snakes bite them. Therefore, we suggest that an initial dose be given as follows: 1 vial for mild envenomation, 2 for moderate envenomation, and 2–3 for severe envenomation. Observation of the clinical course of the affected limbs should then be carried out. If rapid limb swelling (such as swelling progressing across the elbow joint within 6 h) occurs, more antivenom should be added. Twelve hours after the initial administration of antivenom, more antivenom should be administered if the limb swelling continues progressing.

In a previous study, most Taiwan habu or green bamboo viper bite patients were successfully treated by the administration of one vial of antivenom and discharged without complications. The more severe envenomated patients were treated with three vials of antivenom [2]. Some observational studies also concluded that administering one vial was sufficient to treat most patients [20,21]. Although, one study conducted by Chen et al. [22] revealed that a larger dose was needed to treat Taiwan habu/green bamboo viper snakebites. However, another study (which was performed in the same hospital and with almost the same patient population as Chen et al. [5]) told us that the higher antivenom dose might be due to those patients whom surgeons treated receiving a higher FH antivenom dosage (5.9 ± 4.2 vials) than those who were treated by emergency medicine physicians (2.7 ± 1.6 vials). Since a similar outcome was observed in that study [5], it is reasonable to conclude that a smaller antivenom dose may be sufficient to treat Taiwan habu/green bamboo viper bite patients. The Taiwan poison control center recommended using the FH antivenom as one to two vials of antivenom to treat green bamboo viper-bitten patients and two to four vials to treat Taiwan habu-bitten patients [13]. However, there is no description of clinical symptom severity score systems to guide the use of antivenom. In this study, we offer a serum venom antigen level-based clinical severity grading system and use it to guide the administration of antivenom.

### 3.2. The Ability of Antivenom in Binding Venom in Snakebite-Envenomated Patients

According to WHO guidelines [23] for the production, control, and regulation of snake antivenom immunoglobulins, tests such as ED_50_ and LD_50_ were used in preclinical settings to ensure that antivenoms can neutralize venoms. Factors such as the different pathogenic effects of venom toxins and venom pharmacokinetics in humans and laboratory animals have existed. ED_50_ and LD_50_ cannot promise the clinical usefulness of antivenom in treating snakebite patients. Besides, delays between the envenoming bite and antivenom treatment may further complicate if the antivenom is effective in treating venomous snakebite patients. Suggestions have been made on whether some antivenoms bind and neutralize specific venom toxins, and their inability to do this has resulted in antivenom failure in the clinical setting [6]. Fortunately, the FH antivenom had high neutralizing capacities in an animal study. The lethal potencies of the venom of Taiwan habu and green bamboo viper were reduced to 1.8 and 0.9% of the originals, while their hemorrhagic activities were cut down to 0.05% and 0.01% of the crude venom, respectively [10]. This study demonstrated that the FH antivenom could bind and eliminate venom in snakebite patients, thus, together with the results of the previous animal study, providing the base of its usefulness in the clinical setting.

### 3.3. The Clinical Usefulness of Antivenom Therapy

Some studies have demonstrated that antivenom has a controversial role in reducing local tissue swelling [24,25,26]. For example, antivenom in treating *Echis carinatus* snakebites in Nigeria showed even further increases in limb circumference. It increased in swelling extent even after antivenom treatment, and swelling persisted for at least one week after the bite in most cases [25]. A randomized controlled trial of antivenom for the local effects of green pit vipers (*Trimeresurus albolabris* or *T. macrops*) disclosed that intravenous antivenom could accelerate local edema resolution but did not reach clinical significance. Therefore, intravenous antivenom use to reduce green pit viper bites related to limb swelling in Thailand was not recommended [26]. Dart et al. [24] conducted a trial of crotaline polyvalent immune Fab antivenom for treating crotaline snakebites in the United States. The antivenom can reduce the local limb swelling, but local manifestations recur. One of the explanations for this kind of local recurrence is probably due to the short half-life of the Fab antivenom in nature [24]. Lacking the antivenom pharmacokinetics after 6 h of antivenom administration cannot provide us with a rebound of venom in the FH antivenom. The recurrence of envenoming in patients was because of the rapid elimination of Fab antivenoms [27,28]. On the contrary, the FH antivenom is a bivalent antibody (F(ab′)_2_) that may be effective for the complete and prolonged neutralization of intravascular toxins, which have a long half-life in envenomed patients [23,29]. Since there were no clinical recurrence phenomena of the re-progression of limb swelling in patients whose limb swelling resolved within 12 h, we thought the venom rebound phenomenon did not exist in the FH antivenom.

**Figure 3:**
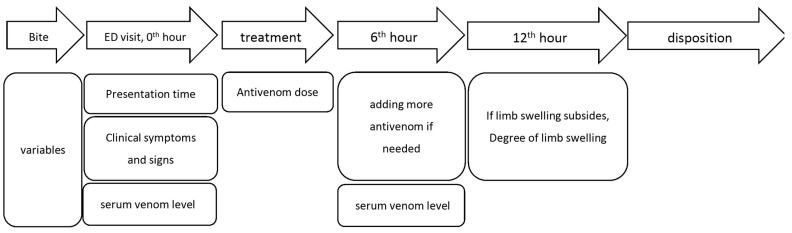
Study flow chart and timeline of data collection.

Since this is not a randomized controlled study, we can only provide indirect evidence that the FH antivenom could effectively reduce limb swelling after Taiwan habu/green bamboo viper bites. In this study, we demonstrated that most enrolled patients (25/33) had their limb swelling subside within 12 h after antivenom treatment. In those who could not have their limbs swelling reduced in 12 h, we thought it was due to inadequate antivenom administration. In Figure 3, we determined that an increase of 1 degree in maximum swelling multiplied the odds of swelling not subsiding within 12 h by an odds ratio of 1.5 (95% CI 1.0–2.0, *p* = 0.03). That is to say, more severe limb swelling was more challenging to reduce. As we demonstrated in Table 3, patients who received adequate antivenom doses had their limbs swelling subside in 12 h more than those who did not receive sufficient antivenom doses. Thus, we thought FH antivenom could be used in treating the limb swelling of Taiwan habu/green bamboo viper snakebite patients if an adequate antivenom dose was provided.

### 3.4. Limitations and Further Perspectives

Our sample size was small. We calculated the power by comparing white blood cell levels between limb swelling subsiding within 12 h or not, and the effect size was 0.89, resulting in a power of 0.8.

Lacking the pharmacokinetics of venoms and antivenoms is a weak point in our study. Further studies to investigate the pharmacokinetics of venoms and antivenoms could help assess antivenom’s usefulness and would be helpful to clarify further the role of FH antivenom in the clinical setting.

The dose suggestion provided in this study should be used cautiously because it was achieved only based on the small patient population. Further studies to investigate the pharmacokinetics of venoms and antivenoms could help us gain more insight into assessing the usefulness of antivenom.

## 4. Conclusions

In conclusion, our data indicate the feasibility of the FH antivenom both in effectively eliminating venom and resolving the affected limb swelling caused by Taiwan habu and green bamboo viper bites.

## 5. Materials and Methods

### 5.1. Enrolment of Patients

This is a prospective, observational study. All patients older than 18-year-old with suspected Taiwan habu/green bamboo viper snakebites who presented to the emergency departments (ED) of Chang Gung memorial hospital from January 2019 to January 2021 were enrolled in the study. Patients who came from another hospital with initial antivenom treatment were included in this study, too. Pregnant women were excluded. If the patient refused to join this study or refused further treatment in ED or after admission, they were excluded.

### 5.2. Study Protocol and Definitions

Demographic variables of age, gender, and snakebite information (the culprit snake, time of biting) were collected. We took photos of patients’ wounds when arriving at the ED and 12 h after or when their affected limbs reached maximum swelling. The patients were asked to identify the culprit snake through a pictorial atlas of the six medically critical venomous snakes in ED. The species of the culprit offending snakes were determined by our previously developed ELISA method later [14].

Figure 3 is the study flow chart and timeline of data collection. When a patient arrived at the ED, the clinical symptoms and signs such as the location and degree of limb swelling and its progression were recorded. The patient’s blood samples were collected for the determination of the serum venom antigen levels, complete cell counts (CBC), prothrombin time (PT), and activated partial thromboplastin time (aPTT) before the administration of antivenom. The serum venom antigen levels before administering antivenom (pre-antivenom serum venom antigen levels) were employed to assess the relationship between serum venom antigen levels and clinical severity. Six hours after administering the antivenom, we collected another blood sample to detect the post-antivenom serum venom antigen levels. The patients with pre-antivenom and post-venom serum venom antigen levels were checked to examine the changes in serum venom antigen levels before and after antivenom.

We defined the presentation time as the time since the patients had been bitten until their visit to ED. The time interval of progression was defined as the time interval between the bite and the limb swelling progressing to the first joint. The clinical outcome measurement was the limb swelling subsiding 12 h after antivenom treatment. When venom enters limbs after snakebites, inflammation begins, and thus the envenomated limb begins to swell soon after the bites. However, because venom-related tissue inflammation is not quickly resolved after the antivenom administration, the swelling limb subsiding would be much delayed even if the venom is neutralized by antivenom. Therefore, we chose if the limb swelling subsided 12 h after antivenom treatment but not 6 h after antivenom administration.

If a patient was referred from another hospital, then there was no blood sample collected for the pre-antivenom serum venom antigen levels if they received antivenom in the first aid hospital. However, the post-antivenom serum venom antigen levels were determined.

### 5.3. The Severity of Local Manifestations of Envenomation

Since there is no consensus on the severity grading for snakebite envenomation in Taiwan, we defined a limb swelling scale (Table S1) modified from a previous publication [30] while considering the length between different joints. Additionally, we then defined the clinical severity of the local manifestations of envenoming as mild, moderate, and severe. For example, if a patient is bitten on the finger and the maximum swelling is confined to the finger, it has a swelling scale of 1 point. If a patient is bitten on the finger and the maximum swelling is up to the wrist, it has a swelling scale of 2 points. However, if a patient is bitten in the wrist and the maximum swelling is up to the elbow, then the swelling scale of 4 points (the point of the wrist is 3, and the elbow is 7; therefore, the swelling scale would be 7–3 = 4). The degree of swelling is defined as follows: mild: 0–2 points; moderate 3–4 points; severe ≥ 5.

### 5.4. Patient Management and the Administration of Antivenom

Antivenom treatment is indicated in patients who receive systemic envenoming or local envenoming with local swelling involving more than half of the bitten limb (in the absence of tourniquet) within 48 h of the bite; swelling after bites on digits, rapid extension of swelling beyond wrist/ankle within a few hours of bites on the hand/foot, and enlarged tender lymph node-draining bitten limbs [31]. If indicated, the choice of antivenom depends on the venomous snake species; therefore, the patients will be asked to identify it through a pictorial chart available in the ED. The species of the culprit offending snakes were determined by the patients’ identification of the snakes and the ELISA-based venom detection method we developed earlier [14]. The ED physicians determined the dose of the administrated antivenom according to the degree of severity as one (mild degree), two (moderate degree), and three vials (severe degree). After administering the antivenom, the patients were monitored in the ED to observe if there was a clinical improvement in their limb swelling. No more antivenom is to be added if the limb swelling is stopped or if there is no progression more than another joint within 6 h (beyond the elbow and knee, respectively, if a patient is bitten in the hand/foot/wrist and ankle). On the contrary, those whose limb swelling progresses beyond another joint within 6 h can be treated in another vial of antivenom. However, if the swelling goes rapidly, then another or more vials of antivenom could be administrated depending on the clinical judgment of the ED physicians.

### 5.5. Detection of Serum Venom Antigen Levels

The venom antigen levels of each victim’s serum were measured by the venom-detected ELISA assay described previously [14]. Briefly, a serum sample (100μL) was added into the microplate coated with capture antibodies recognized hemorrhagic (Taiwan habu and green bamboo viper) venom-specific proteins and incubated for 30 min at room temperature (RT). After washing six times with PBST, 100μL of detection antibodies with HRP labeling, diluted 1:16,000 in PBS, was added onto the microplate and incubated for 30 min. This microplate was washed six times with PBST again. Then, 3,3′5,5′-Tetramethylbenzidine (*TMB*) buffer was added to each well and incubated for 10 min, stopping the reaction by 2N H_2_SO_4_. The absorbance of each well was measured by a SpectraMax M5 microplate reader at a wavelength of 450 nm. The venom antigen levels of each sample were calculated according to the standard curves of crude hemorrhagic venoms. The ELISA for hemorrhagic venoms was determined to be 0.78 ng/mL.

### 5.6. Statistics

For statistical analyses, continuous variables were presented as the mean, standard deviation, or median (q1, q3) according to the normality. We performed chi-squared or Fisher’s exact tests to examine the associations between categorical variables or a small sample of categorical variables among studied groups. Analysis of variance was used to compare the differences in the continuous variables followed by normal distribution between the studied groups. Meanwhile, the Mann–Whitney U test was used to compare the continuous variables that were not normally distributed between the two groups. We also conducted the Spearman correlation coefficients to explore the association between severity and serum concentration variables. Finally, we used logistic regression and a receiver operating characteristic curve (ROC) to observe the association between severity and swelling release within 12 h or not. The Statistical Analysis System (SAS) software version 9.4 (SAS Inc., Cary, NC, USA) was used for the data analysis. A *p*-value of less than 0.05 was considered significant.

## Figures and Tables

**Table 1:** Demographic characteristics.

Variables	Offending Snakes		
	Taiwan Habu n = 14 (30.4%)	Green Bamboo Viper n = 19 (41.4%)	*p*
Age, mean ± SD	60.3 ± 17.9	62.2 ± 14.5	0.74 ^a^
Gender, male, n (%)	6 (42.9)	11 (57.9)	0.39 ^b^
Direct ED admission, n (%)	7 (50.0)	15 (79.0)	0.14 ^c^
Presentation time (min), median (IQR)	105 (40)	117 (64)	0.31 ^d^
Laboratory variables (before antivenom)			
WBC (1000/μL), median (IQR)	9.0 (3.8)	7.6 (4.3)	0.18 ^d^
Segment, median (IQR)	71.5 (12.0)	67.0 (17.5)	0.50 ^d^
Hb (g/dL), median (IQR)	15.1 (3.3)	14.5 (1.6)	0.60 ^d^
PLT (1000/μL), median (IQR)	234 (81.0)	244 (93.0)	0.69 ^d^
Prothrombin time, median (IQR)	1.0 (0.1)	1.0 (0.0)	0.31 ^d^
aPTT, median (IQR)	1.0 (0.1)	1.0 (0.1)	0.26 ^d^
Serum venom level (ng/mL), median (IQR)			
Pre-antivenom Post-antivenom	12.1 (17.9) 0.0 (0.2)	28.2 (130.2) 0.0 (15.4)	0.33 0.82
Time interval of progression (min) ^e^, median (IQR)	120 (204)	150 (335)	0.71 ^d^
Degree of initial swelling, n (%)			0.98 ^c^
Mild (≤2 points) Moderate or severe (>2 points)	10 (76.9) 3 (23.1)	13 (76.5) 4 (23.5)	
Degree of maximum swelling, n (%)			0.31 ^c^
Mild (≤2 points) Moderate (3–4 points) Severe (≥5 points)	5 (35.7) 1 (7.1) 8 (57.1)	7 (36.8) 5 (26.3) 7 (36.8)	
Antivenom dosage			0.36 ^c^
1 vial, n (%) 2 vials, n (%) ≥3 vials, n (%)	4 (28.6) 7 (50.0) 3 (21.4)	10 (52.6) 5 (26.3) 4 (21.1)	
Swelling subsides (12 h), yes, n (%)	9 (64.3)	16 (84.2)	0.24 ^c^

^a^ Two sample *t*-test, ^b^ Chi-square test, ^c^ Fisher’s exact test, ^d^ Mann–Whitney U test; ^e^ degree of initial swelling/time of the presentation time(h).

**Table 2:** Spearman correlation between swelling severity and presentation time, venom antigen levels, and total antivenom doses.

Variables	Initial Limb Swelling	Maximum Limb Swelling
Taiwan habu		
Presentation time	0.14	0.13
Pre-antivenom serum venom antigen levels	0.40	0.93 **
Total vials dose	−0.01	0.70 **
Green bamboo viper		
Presentation time	−0.03	−0.001
Pre-antivenom serum venom antigen levels	0.29	0.71 **
Total vials dose	0.71 **	0.70 **

** *p* < 0.01.

**Table 3:** Comparison between patients with swelling subsiding within 12 h and not.

	Swelling Subsided in 12 h		
Variables	Group A ^#^ (Yes) n = 25 (75.8%)	Group B (No) n = 8 (24.2%)	*p*
Age, mean ± std	61.2 ± 16.5	62.1 ± 14.4	0.32 ^a^
Gender, male, n (%)	13 (52.0)	4 (50.0)	1.00 ^b^
ED admission/referral, admission, n (%)	17 (68.0)	5 (62.5)	1.00 ^c^
Presentation time (min), median (IQR)	114 (59.0)	105 (75.0)	0.93 ^d^
Laboratory variables (before antivenom)			
WBC(1000/μL), median (IQR)	7.6 (3.4)	9.5 (2.7)	0.048 ^d^
Segment, median (IQR)	71.3 (14.1)	70.9 (13.1)	0.22 ^d^
Hb (g/dL), median (IQR)	14.4 (1.7)	15.1 (2.2)	0.22 ^d^
PLT (1000/μL), median (IQR)	245 (78.0)	229 (108)	0.48 ^d^
Prothrombin time, median (IQR)	1.0 (0.0)	1.1 (0.4)	0.26 ^d^
aPTT, median (IQR)	1.0 (0.1)	1.0 (0.1)	0.77 ^d^
Serum venom level (ng/mL), median (q1, q3)			
Taiwan habu			
Pre-antivenom Post-antivenom	7.1 (11.9) 0 (0.2)	21.7 (14.4) 0 (0.1)	0.27 ^d^ 0.94 ^d^
Green bamboo viper			
Pre-antivenom Post-antivenom	14.4 (83.4) 0 (1.6)	169.8 (363.1) 7.7 (15.4)	0.12 ^d^ 0.84 ^d^
Time interval of progression (min)	180 (181)	100 (408)	1.00 ^d^
Degree of initial swelling, n (%)			
Mild (≤2 points) Moderate (>2 points)	19 (82.6) 4 (17.4)	4 (57.1) 3 (42.9)	0.31 ^c^
Maximum swelling degree			0.02 ^c^
Mild (≤2 points) Moderate (3–4 points) Severe (≥5 points)	12 (48.0) 4 (16.0) 9 (36.0)	0 (0.0) 2 (25.0) 6 (75.0)	
Antivenom dosage			
1 vial, n (%) 2 vials, n (%) ≥3 vials, n (%)	12 (48.0) 9 (36.0) 4 (16.0)	2 (25.0) 3 (37.5) 3 (37.5)	0.38 ^c^

^#^ Group A: patients had their swelling subside within 12 h after receiving antivenom; group B: patients whose swelling did not subside within 12 h after receiving antivenom; ^a^ Two sample *t*-test, ^b^ Chi-square test, ^c^ Fisher’s exact test, and ^d^ Wilcoxon rank sum test.

**Table 4:** FH dose suggestions to treat Taiwan habu and green bamboo viper bites.

Taiwan Habu (n = 14)					
Max Swelling	Subside in 12 h	Vial			Suggestion
		1	2	≥3	
≤2 points mild	yes	3 ^#^	2	--	1–2 vials are sufficient
	no	0	0	--	
3–4 points Moderate	yes	0	--	--	---
	no	0	1	--	
≥5 points severe	yes	1	2	1	May need at least 2–3 vials
	no	0	2	2	
**Green Bamboo Viper (n = 19)**					
**Max Swelling**	**Subside in 12 h**	**Vial**			**Suggestion**
		**1**	**2**	**3**	
≤2 points mild	yes	6	1	--	1 is sufficient
	no	0	0	--	
3–4 points Moderate	yes	2	2	--	2 vials are sufficient
	no	1	0	--	
≥5 points severe	yes	0	2	3	May need at least 2–3 vials
	no	1	0	1	

^#^ means three patients of mild envenomation were treated with one vial of antivenom and their limb swelling subsided within 12 h.

## Data Availability

The data presented in this study are available in this article or Supplementary Materials.

## Supplementary Materials

The following supporting information can be downloaded at: https://www.mdpi.com/article/10.3390/toxins14110794/s1, Table S1: Limb swelling scale and definition of clinical severity.
