# Supplementary material for: The Clinical Usefulness of Taiwan Bivalent Freeze-Dried Hemorrhagic Antivenom in Protobothrops mucrosquamatus- and Viridovipera stejnegeri-Envenomed Patients
Source: Toxins (Basel). 2022 Nov 15;14(11):794. doi: 10.3390/toxins14110794 (PMC9699225; doi:10.3390/toxins14110794)
Supplement: Supplementary file 1 [file toxins-14-00794-s001.zip › toxins-1959167-supplementary.pdf]

Table S1. Limb swelling scale and definition of clinical severity.

Upper limb	finger	hand	wrist	forearm	elbow	Upper arm	shoulder	
points	1	2	3	5	7	9	11	
Lower limb	toe	foot	ankle	Lower leg	knee	thigh	buttock	waist
points	1	2	3	6	9	12	15	16

Degrees of swelling are as following: mild: 0-2 points; moderate 3-4 points; severe ≥ 5
